# Enhancement of Secondary Cell Wall Formation in Poplar Xylem Using a Self-Reinforced System of Secondary Cell Wall-Related Transcription Factors

**DOI:** 10.3389/fpls.2022.819360

**Published:** 2022-03-14

**Authors:** Yoshimi Nakano, Hitoshi Endo, Lorenz Gerber, Chiaki Hori, Ayumi Ihara, Masayo Sekimoto, Tomoko Matsumoto, Jun Kikuchi, Misato Ohtani, Taku Demura

**Affiliations:** ^1^Graduate School of Science and Technology, Nara Institute of Science and Technology, Ikoma, Japan; ^2^Department of Forest Genetics and Plant Physiology, Umeå Plant Science Centre, Swedish University of Agricultural Sciences, Umeå, Sweden; ^3^RIKEN Center for Sustainable Resource Science, Yokohama, Japan; ^4^Department of Integrated Biosciences, Graduate School of Frontier Sciences, The University of Tokyo, Kashiwa, Japan

**Keywords:** secondary cell wall, xylem, transcription factor, AtVND7, AtSND1, AtMYB46, hybrid aspen

## Abstract

The secondary cell wall (SCW) in the xylem is one of the largest sink organs of carbon in woody plants, and is considered a promising sustainable bioresource for biofuels and biomaterials. To enhance SCW formation in poplar (*Populus* sp.) xylem, we developed a self-reinforced system of SCW-related transcription factors from *Arabidopsis thaliana*, involving VASCULAR-RELATED NAC-DOMAIN7 (VND7), SECONDARY WALL-ASSOCIATED NAC-DOMAIN PROTEIN 1/NAC SECONDARY WALL THICKENING-PROMOTING FACTOR3 (SND1/NST3), and MYB46. In this system, these transcription factors were fused with the transactivation domain VP16 and expressed under the control of the *Populus trichocarpa CesA18* (*PtCesA18*) gene promoter, creating the chimeric genes *PtCesA18pro::AtVND7:VP16*, *PtCesA18pro::AtSND1:VP16*, and *PtCesA18pro::AtMYB46:VP16*. The *PtCesA18* promoter is active in tissues generating SCWs, and can be regulated by AtVND7, AtSND1, and AtMYB46; thus, the expression levels of *PtCesA18pro::AtVND7:VP16*, *PtCesA18pro::AtSND1:VP16*, and *PtCesA18pro::AtMYB46:VP16* are expected to be boosted in SCW-generating tissues. In the transgenic hybrid aspens (*Populus tremula × tremuloides* T89) expressing *PtCesA18pro::AtSND1:VP16* or *PtCesA18pro::AtMYB46:VP16* grown in sterile half-strength Murashige and Skoog growth medium, SCW thickening was significantly enhanced in the secondary xylem cells, while the *PtCesA18pro::AtVND7:VP16* plants showed stunted xylem formation, possibly because of the enhanced programmed cell death (PCD) in the xylem regions. After acclimation, the transgenic plants were transferred from the sterile growth medium to pots of soil in the greenhouse, where only the *PtCesA18pro::AtMYB46:VP16* aspens survived. A nuclear magnetic resonance footprinting cell wall analysis and enzymatic saccharification analysis demonstrated that *PtCesA18pro::AtMYB46:VP16* influences cell wall properties such as the ratio of syringyl (S) and guaiacyl (G) units of lignin, the abundance of the lignin β-aryl ether and resinol bonds, and hemicellulose acetylation levels. Together, these data indicate that we have created a self-reinforced system using SCW-related transcription factors to enhance SCW accumulation.

## Introduction

In recent years, mounting environmental problems, such as global warming from fossil fuels, have increased the importance of sustainable and carbon-neutral bioresources. Lignocellulosic biomass, the most abundant above-ground bioresource, is found in the secondary cell walls (SCWs) of xylem tissues. To improve the availability and use of lignocellulosic biomass, the molecular mechanisms of SCW formation have been actively studied. In the last 2 decades especially, our understanding of the key transcriptional regulators for SCW formation has greatly expanded (Zhong et al., 2010a; Nakano et al., 2015; Ohtani and Demura, 2019; Kamon and Ohtani, 2021). One of the most important findings is the identification of key transcriptional factors of SCW formation; in vascular plants, a specific group of NAC (NAM/ATAF/CUC) family transcription factors, called VNS [VASCULAR-RELATED NAC-DOMAIN (VND), NAC SECONDARY WALL THICKENING PROMOTING FACTOR (NST)/SECONDARY WALL-ASSOCIATED NAC DOMAIN1 (SND), and SOMBRERO (SMB)-related] proteins, were shown to function as master regulators of SCW formation, activating all of the events required for SCW formation in *A. thaliana* (Arabidopsis; Kubo et al., 2005; Mitsuda et al., 2005, 2007; Zhong et al., 2006, 2010b; Yamaguchi et al., 2008, 2011; Ohashi-Ito et al., 2010; Ohtani et al., 2011; Xu et al., 2014; Akiyoshi et al., 2020). Additionally, downstream of the VNS proteins, the MYB transcription factors, such as Arabidopsis MYB46 and MYB83, function to upregulate the expression of genes encoding SCW-related enzymes as secondary master regulators of xylem cell formation (Zhong et al., 2007; Ko et al., 2009, 2014; McCarthy et al., 2009, 2010; Nakano et al., 2010). The NAC–MYB-based transcriptional network of SCW formation is widely conserved among land plants (Zhong et al., 2010a; Xu et al., 2014; Nakano et al., 2015; Bowman et al., 2017; Ohtani et al., 2017a), suggesting that these NAC and MYB transcription factors would be effective targets for modifying the quantity and quality of lignocellulosic biomass.

Phylogenetic tree analysis indicates that three subgroups can be recognized within VNS proteins; VND, SND/NST, and SMB subgroups (Nakano et al., 2015; Bowman et al., 2017; Ohtani et al., 2017a; Akiyoshi et al., 2020). In Arabidopsis, VND and SND/NST subgroups regulate the differentiation of xylem vessels and xylem fibers, respectively (Kubo et al., 2005; Mitsuda et al., 2005, 2007; Zhong et al., 2006, 2010b; Yamaguchi et al., 2008, 2011). It has been shown that cell wall characteristics are different between xylem vessel cells and fiber cells, such as syringyl (S)/guaiacyl (G) ratio of lignin subunits (Saito et al., 2012). Therefore, the regulatory targets of SCW-related genes should be different between VND and SND/NST subgroups; indeed, the transcriptome analysis of Arabidopsis cells carrying the inducible overexpression system of VND or SND/NST revealed that the genes for lignin monomer biosynthesis could be more strongly induced by SND/NST proteins than by VND proteins (Ohashi-Ito et al., 2010; Zhong et al., 2010b; Yamaguchi et al., 2011). In addition, a part of SCW-biosynthetic genes, for example SCW-specific cellulose synthase genes *CesA4* and *CesA8*, are common targets of VNS and MYB proteins (Zhong et al., 2007, 2010b; Ko et al., 2009, 2014; McCarthy et al., 2009, 2010; Nakano et al., 2010; Ohashi-Ito et al., 2010; Yamaguchi et al., 2011). Interestingly, in Arabidopsis, most of SCW-biosynthetic genes would be mainly regulated by the MYB46 and MYB83 proteins, since the SCW deposition in xylem cells is severely defective in the *myb46 myb83* double mutant (McCarthy et al., 2009), and the *cesa* mutant phenotype could not be rescued when the MYB46-regulatory cis element was mutated in the promoter controlling *CesA* genes (Kim et al., 2013). These findings collectively suggest that each SCW-related transcription factor would differently regulate the characteristics of SCW in xylem tissues.

The effects of *VNS* and *MYB* overexpression have already been reported in several plant species, including Arabidopsis, rice (*Oryza sativa*), and a hybrid aspen (*Populus tremula × tremuloides*; Kubo et al., 2005; Mitsuda et al., 2005, 2007; Zhong et al., 2006, 2007, 2011; McCarthy et al., 2010; Ohtani et al., 2011; Valdivia et al., 2013; Yoshida et al., 2013; Xu et al., 2014; Endo et al., 2015; Akiyoshi et al., 2020). These reports showed that the continuous overexpression of *VNS* or *MYB* under the control of the cauliflower mosaic virus *35S* promoter resulted in the ectopic deposition of SCWs, resulting in the inhibition of plant growth. Moreover, the transgenic expression of *VNS* genes from other species showed a higher induction activity of ectopic SCW deposition than the overexpression of the gene from the same species (Kubo et al., 2005; Ohtani et al., 2011; Sakamoto et al., 2016). These observations led us to develop a new hypothesis that the enhanced xylem tissue–specific expression of heterologous *VNS* and *MYB* genes would be an effective strategy for enhancing SCW deposition in the xylem without negatively impacting plant growth.

In order to test the hypothesis described above, we designed a self-reinforced system of SCW-related transcription factors from Arabidopsis: AtVND7, AtSND1/AtNST3, and AtMYB46. In this system, to make an artificial positive feedback loop (Yan et al., 2012), the transcription factors were fused with the VP16 transcription activation domain to enhance their function (Yamaguchi et al., 2010). In addition, they were expressed under the control of the *Populus trichocarpa CesA18* (*PtCesA18*) promoter, which is active and the gene is expressed in xylem tissue (Suzuki et al., 2006). The resultant chimeric genes, *PtCesA18pro::AtVND7:VP16*, *PtCesA18pro::AtSND1:VP16*, and *PtCesA18pro::AtMYB46:VP16*, were introduced into the hybrid aspen (*P. tremula × tremuloides* T89). The phenotypic analysis showed that our strategy could work to enhance SCW deposition in xylem tissues in *PtCesA18pro::AtSND1:VP16* and *PtCesA18::AtMYB46:VP16*. We also found that only transgenic plants expressing *PtCesA18pro::AtMYB46:VP16* were able to grow in pots of soil in the greenhouse, while *PtCesA18pro::AtVND7:VP16* and *PtCesA18pro::AtSND1:VP16* could grow only in a sterile half-strength Murashige and Skoog (MS) medium. The lignocellulosic property in the xylem tissues was altered in the *PtCesA18pro::AtMYB46:VP16* line 17 with the highest expression of *AtMYB46*. Together, these data collectively demonstrate the practicality of using a self-reinforced system of SCW-related transcription factors to enhance lignocellulose accumulation and SCW deposition, especially for the second master regulator of xylem cell formation (AtMYB46) in woody plants.

## Materials and Methods

### Plant Materials and Growth Conditions

The promoter sequence of the *PtCesA18* gene was amplified from the genome of black cottonwood (*Populus trichocarpa*). Hybrid aspen (*P. tremula* × *P. tremuloides*, T89) was used for the transformation, according to the methods described by Ohtani et al. (2011). The plants were maintained and propagated aseptically on a medium containing a half-strength Murashige and Skoog (MS) salt mixture (Duchefa Biochemie, Haarlem, The Netherlands; pH 5.8) under a photoperiod of 16 h light/8 h dark at 23°C (Ohtani et al., 2011; Hori et al., 2020). For the analysis of growth and xylem lignocellulose, the transgenic plants were acclimated to the pots of soil in the greenhouse and grown under a 16-h light/8-h dark photoperiod at 23°C (Ohtani et al., 2011; Hori et al., 2020). To clone the transcription factor genes, total RNAs were extracted from 7-day-old *A. thaliana* (ecotype Columbia) seedlings grown in half-strength MS medium containing 0.5% (w/v) sucrose, as described by Nakano et al. (2010).

### Vector Construction and Generation of Transgenic Aspens

An approximately 3-kbp promoter sequence of *PtCesA18* (also known as *PtrCesA1A/PtCesA3-2/PtiCesA8-B* or eugene3.00040363; Taylor et al., 2003; Suzuki et al., 2006; Kumar et al., 2009), which was most homologous to a *Populus tremuloides CesA* that was previously characterized as a xylem-specific cellulose synthase gene (Wu et al., 2000), was amplified using PCR from the genomic DNA isolated from *Populus trichocarpa* shoots as described by Ohtani et al. (2011), and then cloned into pENTR™/D-TOPO™ (Thermo Fisher Scientific, Waltham, MA, United States). The coding regions (excluding the stop codon) of *AtVND7* (AT1G71930), *AtSND1* (AT1G32770), and *AtMYB46* (AT5G12870) were also PCR-amplified from the cDNAs prepared from the total RNAs of the *Arabidopsis* seedlings, as described by Nakano et al. (2010), and then cloned into pENTR™/D-TOPO™ (Thermo Fisher Scientific, Waltham, MA, United States). The *HYGROMYCIN PHOSPHOTRANSFERASE* gene and *NOS* terminator of the pSMAH621 vector (Kubo et al., 2005) were replaced with the *NEOMYCIN PHOSPHOTRANSFERASE II* (*NPTII*) gene and the Arabidopsis heat shock protein terminator (*AtHSP* ter), respectively, to increase mRNA expression (Nagaya et al., 2010). To generate the construction of *PtCesA18pro::GUS, PtCesA18* promoter sequence was inserted into the *Sma* I site located the upstream of *GUS* gene in the modified pSMAH621 containing *NPTII* and *AtHSP* ter. In addition, the *35S* promoter and the *GUS* gene were removed from pSMAH621 using restriction enzymes, and the sequence of the human herpes virus–derived VP16 transcriptional activation domain was inserted, to obtain the pSMAH-VP16-Thsp vector.

**Figure 1:**
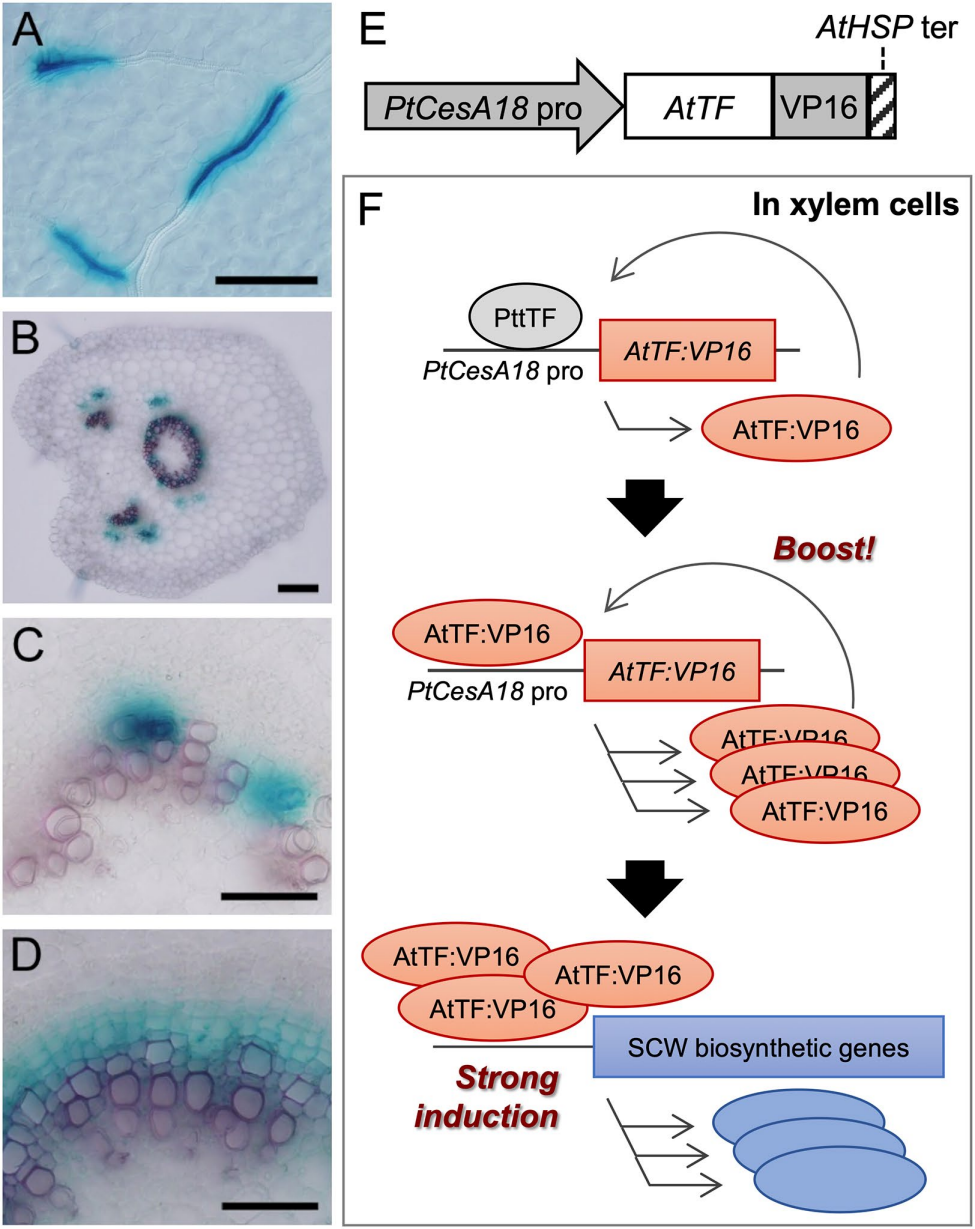
The concept of the self-reinforced system with secondary cell wall (SCW)-related transcription factors. **(A–D)** The promoter activity of the *Populus trichocarpa CesA18* (*PtCesA18*) gene in the hybrid aspen (*Populus tremula × tremuloides* T89). The activity was determined using the *PtCesA18pro::GUS* reporter in the leaf **(A)**, the petiole **(B)**, the elongating third internode **(C)**, and the elongating fifth internode **(D)**. GUS signals were observed in the developing leaf veins **(A)**, xylem and phloem fibers in the petiole **(B)**, and developing xylem tissues **(C,D)**. Bars = 100 µm in **(A,B)**, 50 µm in **(C,D)**. **(E)** Schematic diagram of the construct used for the self-reinforced system. In this system, a gene encoding an *Arabidopsis thaliana* transcription factor (TF) is designed to be expressed under the control of the *PtCesA18* promoter, forming a fusion protein with VP16, a transcriptional activation domain derived from the human herpes virus. The transcription of transgene is terminated by the Arabidopsis heat shock protein terminator (*AtHSP* ter; Nagaya et al., 2010). **(F)** Mode of action of the self-reinforced system with SCW-related transcription factors. (Upper) In the xylem cells of hybrid aspen (*Populus tremula × tremuloides*), the *PtCesA18* promoter is activated by an endogenous transcription factor (PtTF), resulting in the generation of AtTF:VP16, which is introduced by the construct described in **(E)**. (Middle) The generated AtTF:VP16 can strongly activate the *PtCesA18* promoter activity to boost the synthesis of AtTF:VP16 in a self-reinforcing manner. (Lower) The generated AtTF:VP16 fusion protein subsequently induces endogenous SCW-related genes. TF, transcription factor; At, *Arabidopsis thaliana*; Pt, *Populus trichocarpa*; and Ptt, *Populus tremula × tremuloides*.

To generate the sequence corresponding to *PtCesA18pro::AtVND7*, *PtCesA18pro::AtSND1*, or *PtCesA18pro::AtMYB46*, the sequences of *PtCesA18pro* and the coding regions of *AtVND7*, *AtSND1*, and *AtMYB46* were first independently amplified by PCR with a set of primers containing the overlapped sequences between the *PtCesA18pro* sequence and the coding regions of *AtVND7*, *AtSND1*, or *AtMYB46*, and a primer containing the recognition site of a restriction enzyme. The PCR products were used as the templates for a second PCR to amplify the sequences of *PtCesA18pro::AtVND7*, *PtCesA18pro::AtSND1*, or *PtCesA18pro::AtMYB46*, using the primer sets comprising PtCesA18p-EV_F and VND7(−stop)_Avr_R, SND1(−stop)_Avr_R, or MYB46(−stop)_Avr_R. The resultant PCR products were cloned into the pGEM T-easy cloning vector (Promega, Madison, WI, United States), and the sequences of *PtCesA18pro::AtVND7*, *PtCesA18pro::AtSND1*, or *PtCesA18pro::AtMYB46* were cut out with E*co*RV (TaKaRa Bio, Kusatsu, Japan) and *Avr*II (TaKaRa Bio). The pSMAH-VP16-Thsp vector was treated with *Bam*HI (TaKaRa Bio), followed by a treatment with Klenow Fragment (TaKaRa Bio) to generate the blunt ends. The resultant sample was subsequently treated with *Xba*I, and then used for the ligation with the *PtCesA18pro::AtVND7*, *PtCesA18pro::AtSND1*, or *PtCesA18pro::AtMYB46* fragments, to obtain the plant expression vector for the expression of *PtCesA18pro::AtVND7:VP16*, *PtCesA18pro::AtSND1:VP16*, or *PtCesA18pro::AtMYB46:VP16* (Figure 1E). The plasmid was electroporated into the *Agrobacterium tumefaciens* (GV3101::pMP90), and each individual clone was used for the transformation of hybrid aspen (Ohtani et al., 2011). The successful insertion of the transgene in each transgenic aspen was confirmed by PCR with the extracted genomic DNA samples using specific primer sets. The primer sequence information is indicated in Supplementary Table 1.

### Total RNA Extraction From Aspen Samples

Stem tissues of transgenic aspens were collected and frozen in liquid nitrogen, then ground with a mortar and pestle. A 600-μl aliquot of extraction buffer [2% (w/v) cetyltrimethylammonium bromide, 2.5% (w/v) polyvinylpolypyrrolidone 40, 2 M NaCl, 100 mM Tris–HCl (pH 8.0), 25 mM EDTA (pH 8.0), and 2% (v/v) 2-mercaptoethanol] was added to each 100–150-mg sample, and the mixture was homogenized by vortexing. After incubating the samples at 65°C for 10 min, an equal volume of chloroform:isoamylalcohol (24:1) was added to the mixture, followed by vigorous shaking. Subsequently, the samples were centrifuged at 11,000 *g* for 10 min at 4°C, after which the aqueous phase was transferred into a new tube. The extraction using chloroform:isoamylalcohol and the centrifugation were repeated two times in total. LiCl solution (to a final concentration of 3 M) was added to the collected aqueous parts, and the mixture was incubated overnight at 4°C. The samples were centrifuged at 15,000 *g* for 20 min at 4°C to precipitate the RNA fractions, and the precipitated RNA fractions were purified using RNeasy Mini Kit (QIAGEN, Venlo, The Netherlands) to obtain the total RNA samples. The RNA samples were kept at −80°C until required.

### Reverse Transcription-Quantitative PCR

The total RNA samples from transgenic aspens were treated with RQ1 RNase-Free DNase (Promega) following the manufacturer’s instructions. A 2-μg aliquot of DNase-treated RNA was used to synthesize cDNA with the Transcriptor First Strand cDNA Synthesis Kit (Roche Diagnostics, Basel, Switzerland) using oligo dT_(18)_ primers (Promega). The cDNA samples were kept at −20°C until required. The amounts of mRNAs were quantified using LightCycler 480 SYBR Green I Master (Roche), gene-specific primer sets (Supplementary Table 1), and LightCycler 480 Real-Time PCR System (Roche). The expression levels of the analyzed genes were normalized against the expression level of *ELONGATION INITIATION FACTOR 4a* (*ELF4A*; Ohtani et al., 2011; Hori et al., 2020; Akiyoshi et al., 2021).

### Transmission Electron Microscopy

The 10th elongated internodes of the stems were harvested and fixed in 0.05 M sodium phosphate buffer (pH 7.2) containing 1.25% (v/v) glutaraldehyde and 2% (w/v) paraformaldehyde, then fixed with 1% (w/v) osmium tetroxide. The fixed samples were washed with 8% (w/v) sucrose solution and dehydrated using an ethanol series [25, 60, 80, 99, and 100% (v/v)]. The ethanol was replaced with propylene oxide, and thereafter the propylene oxide was replaced with Spurr’s resin (Polysciences, Warrington, PA, United States). Subsequently, the samples were fully embedded in Spurr’s resin. Sections (80 µm thickness) were cut and post-stained with lead stain of Sato (1968) and observed using a transmission electron microscopy (TEM; HITACHI H-7100, HITACHI High-Tech, Tokyo, Japan). The area of the cell walls was calculated by subtracting the area of the cell region surrounded by the plasma membrane from the area of entire cell including the cell wall, using ImageJ.^1^ The statistical analysis was performed using Tukey–Kramer test (*p* < 0.05) in R.^2^

### Nuclear Magnetic Resonance Footprinting Analysis of Transgenic Aspen Stem Tissues

The method for nuclear magnetic resonance (NMR) footprint analysis was described by Hori et al. (2020) and Akiyoshi et al. (2021). The stem samples (about 10-cm in length) were collected from transgenic aspens grown in the greenhouse, freeze-dried, and debarked, then ground in an automill (TK-AM7; Tokken, Kashiwa, Japan) for 10 min. The samples were then further ground in a Pulverisette 5 ball mill (Fritsch, Idar-Oberstein, Germany) for 12 h. Next, 30 mg of powdered sample was dissolved in dimethyl sulfoxide (DMSO)-d_6_:pyridine-d5 (4:1) and heated at 50°C for 30 min with shaking at 14,000 rpm in a Thermomixer Comfort (Eppendorf, Hamburg, Germany). After centrifugation at 15,000 rpm for 5 min, the supernatant was transferred into NMR tubes for analysis. Same supernatant samples were used for Pyrolysis-GC/MS analysis (section “Pyrolysis-GC/MS Analysis”).

Nuclear magnetic resonance spectra were collected on an Avance II HD-700 instrument (Bruker, Billerica, MA, United States) with a 5-mm cryoTCI probe. For the ^1^H and ^13^C analyses, 700.130 and 176.06 MHz resonance frequencies were used, respectively. The temperature for all NMR samples was kept at 318 K. Chemical shifts were referenced to the methyl group of DMSO-d_6_ at ^1^H = 2.49 ppm and ^13^C = 39.5 ppm. The echo/antiecho gradient selections were used for the collection of 2D ^1^H-^13^C hetero-nuclear single quantum coherence (HSQC) spectra. A total of 51 regions of interest (ROI) were identified based on previously assigned chemical shifts (Komatsu and Kikuchi, 2013; Watanabe et al., 2014; Mori et al., 2015). The protocols described in Mansfield et al. (2012) and in Tsuji et al. (2015) were used to quantify HSQC. The peak intensity of pyridine-d5 used for the normalization. The statistical analysis was performed using Tukey–Kramer test (*p* < 0.05) in R.

### Pyrolysis-GC/MS Analysis

The 8-μl of the cell wall extraction sample described in the section “Nuclear magnetic resonance footprinting analysis of transgenic aspen stem tissues” was dropped on pyrofoil F500 (500°C; Japan Analytical Industry Co., Japan). Pyrolysis GC–MS analysis was performed using a Curie Point Pyrolyzer JPS-900 (Curie Point Pyrolyzer, Automated Model, Japan Analytical Industry Co., Ltd., Japan) connected directly to a gas chromatograph system 7890B GC System (Agilent, United States). Pyrolysis was performed using the following conditions; 280°C oven temperature, 280°C needle temperature, and 500°C pyrolysis for 5 s. The pyrolysates were then transferred to the gas chromatograph (5977A MSD, Agilent, United States) equipped with Agilent J&W DB-1 MS column (60 m × 0.25 mm × 0.25 μm, Agilent, United States), and analyzed under the following conditions; carrier, helium; injector temperature, 280°C; split ratio, 100:1; mass range, 50–300; column temperature, heat up from 40 to 280°C at a rate of 4°C/min, and then keep 280°C for 5 min.

Pyrolysis GC–MS data were analyzed based on the methods described by Gerber et al. (2012), Pinto et al. (2012), and Gerber et al. (2016). Briefly, the raw pyrolysis GC–MS data were converted to the NetCDF format by the software Agilent Chemstation Data Analysis, and then the smoothing and alignment of chromatograms were performed (Gerber et al., 2012). After the background subtraction, the Multivariate Curve-Resolution by Alternate Regression (MCR-AR) analysis was performed, to deconvolute the chromatographic and mass spectral profiles. Based on the annotation information of each peak by Gerber et al. (2012), we computed the values for carbohydrate:lignin (C/L) ratio and lignin S/G ratio.

### Enzymatic Saccharification Analysis

The powdered stem samples were treated with a mixture of cellulase from *Trichoderma reesei* ATCC 26921 (Sigma-Aldrich, Merck KgaA, Darmstadt, Germany) and cellobiase from *Aspergillus niger* (Sigma-Aldrich, Merck KgaA) for 24 h, according to the methods of Okubo-Kurihara et al. (2016) and Ohtani et al. (2017b). The supernatant was collected after centrifugation, after which 0.1 M NaOH solution was added to stop the reaction. The released glucose and xylose were measured with a “Glucose CII-Test” (FUJIFILM Wako Pure Chemical Corporation, Osak, Japan) and a “D-Xylose Assay Kit” (Megazyme, Bray, Ireland), respectively.

## Results and Discussion

### Design of the Self-Reinforced System of SCW-Related Transcription Factors

First, we examined the promoter activity of *P. trichocarpa CesA18* (*PtCesA18*), by generating the transgenic *PtCesA18pro:GUS*line of hybrid aspen (*P. tremula × tremuloides* T89; Figures 1A–D). GUS signals were detected in developing xylem vessel cells in the leaf veins (Figure 1A), developing xylem and phloem fiber cells in the petiole (Figure 1B), developing vessel cells in the primary xylem of young internodes (Figure 1C), and developing xylem and fiber cells in the secondary xylem of the older internodes (Figure 1D). These observations indicate that *PtCesA18* promoter activity is tightly associated with SCW formation, as expected; thus, we decided to use the *PtCesA18* promoter for the self-reinforced system of SCW-related transcription factors (Figures 1E,F).

In our self-reinforced system, the transcription factors associated with SCW formation are expressed under the control of the *PtCesA18* promoter, as a fusion protein with the human herpes virus–derived transcriptional activation domain VP16 (Figure 1E). The transcription factor genes used in the present work were *AtSND1*, *AtVND7*, and *AtMYB46*, which are master regulators of SCW formation in *A. thaliana* (Kubo et al., 2005; Mitsuda et al., 2005, 2007; Zhong et al., 2006, 2007; Ko et al., 2009, 2014; Nakano et al., 2015; Ohtani and Demura, 2019; Kamon and Ohtani, 2021). The key feature of this self-reinforced system is that the *PtCesA18* promoter can be upregulated by AtSND1, AtVND7, and AtMYB46, since these transcription factors can recognize and bind their cis-regulatory elements, which are highly conserved among each ortholog (Zhong et al., 2010a; Ohtani et al., 2011; Kim et al., 2012; Zhong and Ye, 2012). First, in the xylem cells, the *PtCesA18* promoter activity is upregulated by endogenous VNS and MYB functions, resulting in the induction of *AtSND1:VP16*, *AtVND7:VP16*, or *AtMYB46:VP16* expression. Subsequently, these induced AtSND1:VP16, AtVND7:VP16, or AtMYB46:VP16 proteins can bind and upregulate the *PtCesA18* promoter to generate large amounts of *AtSND1:VP16*, *AtVND7:VP16*, and *AtMYB46:VP16* mRNAs. We can expect that our constructs will result in the high production of AtSND1:VP16, AtVND7:VP16, and AtMYB46:VP16 proteins, facilitating the strong induction of the SCW biosynthetic genes, as the combined effects of *PtCesA18* promoter and transcriptional activation domain VP16 (Figure 1F).

### Generation of Transgenic Plants Containing the Self-Reinforced System of SCW-Related Transcription Factors

**Figure 2:**
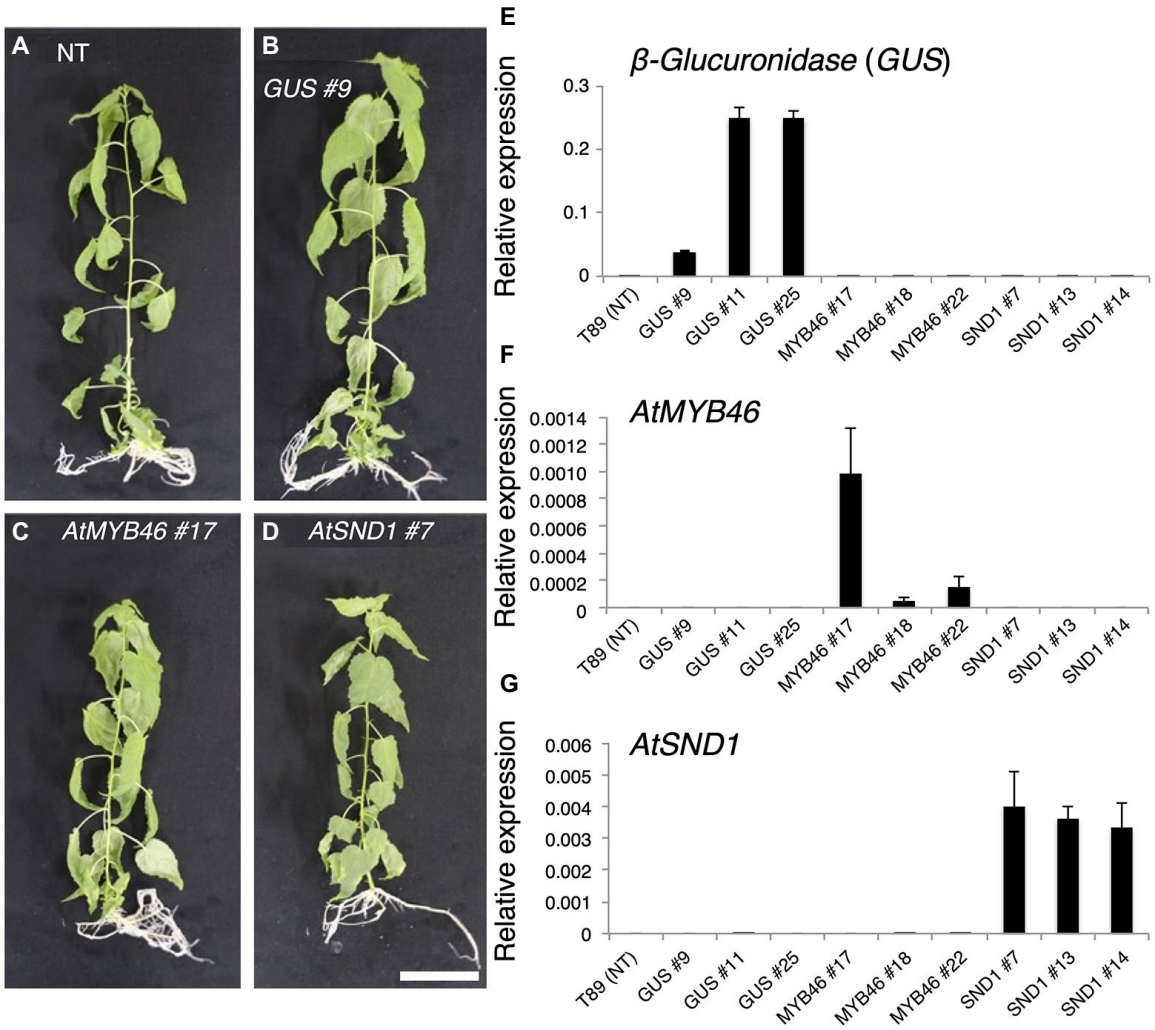
Generation of transgenic aspens carrying *PtCesA18pro::AtMYB46:VP16*, *PtCesA18pro::AtSND1:VP16*, or *PtCesA18pro::AtVND7:VP16*. **(A–D)** Transgenic aspens grown in a sterile half-strength Murashige and Skoog growth medium. Typical views of the non-transgenic (NT) plant (T89; **A**) and plants carrying the *PtCesA18pro::GUS* (*GUS*) line 9 **(B)**, *PtCesA18pro::AtMYB46:VP16* (*AtMYB46*) line 17 **(C)**, or *PtCesA18pro::AtSND1:VP16* (*AtSND1*) line 7 **(D)**. Bar = 5 cm. **(E–G)** Expression levels of the introduced genes, *β-Glucuronidase* (*GUS*; **E**), *AtMYB46* **(F)**, and *AtSND1* **(G)** in the transgenic lines, as quantified using Reverse Transcription-Quantitative PCR (RT-qPCR) analysis. The data are displayed as means ± SD (*n* = 3).

We introduced the chimeric genes *PtCesA18pro::AtSND1:VP16*, *PtCesA18pro::AtVND7:VP16*, and *PtCesA18pro::AtMYB46:VP16*, or the vector control *PtCesA18pro::GUS*, into the hybrid aspen *P. tremula × tremuloides* line T89 (Figures 2A–D, Supplementary Figure 1). As the results, 13, 11, 16, and 14 independent lines of *PtCesA18pro::AtSND1:VP16*, *PtCesA18pro::AtVND7:VP16*, *PtCesA18pro::AtMYB46:VP16*, and *PtCesA18pro::GUS*, respectively, were established. All *PtCesA18pro::AtMYB46:VP16* or *PtCesA18pro::AtSND1:VP16* were indistinguishable from the non-transgenic aspen and control transgenic line *PtCesA18pro::GUS* in terms of their size and morphology when grown in the sterile half-strength MS medium (Figures 2A–D); however, all transgenic *PtCesA18pro::AtVND7:VP16* plants showed severe growth defects (Supplementary Figure 1). The stem sections of the *PtCesA18pro::AtVND7:VP16* lines indicated no clear secondary growth in the xylem and collapsed primary xylem cells (Supplementary Figure 1). It is possible that the decrease of water transport ability and mechanical strength of the stem tissues by the reduction in xylem cells would lead to growth defects in the *PtCesA18pro::AtVND7:VP16* plants. There is a clear difference in transcriptional activation activity between AtVND7 and AtSND1; AtVND7 can strongly induce programmed cell death (PCD)-related genes as a master regulator of xylem vessel cell differentiation, which include PCD process, while AtSND1 possesses a relatively low ability to do so since AtSND1 functions in fiber cell differentiation (Kubo et al., 2005; Ohashi-Ito et al., 2010; Zhong et al., 2010b; Yamaguchi et al., 2011) Thus, the results of the *PtCesA18pro::AtVND7:VP16* lines might reflect this strong activity of AtVND7 to induce PCD.

**Figure 3:**
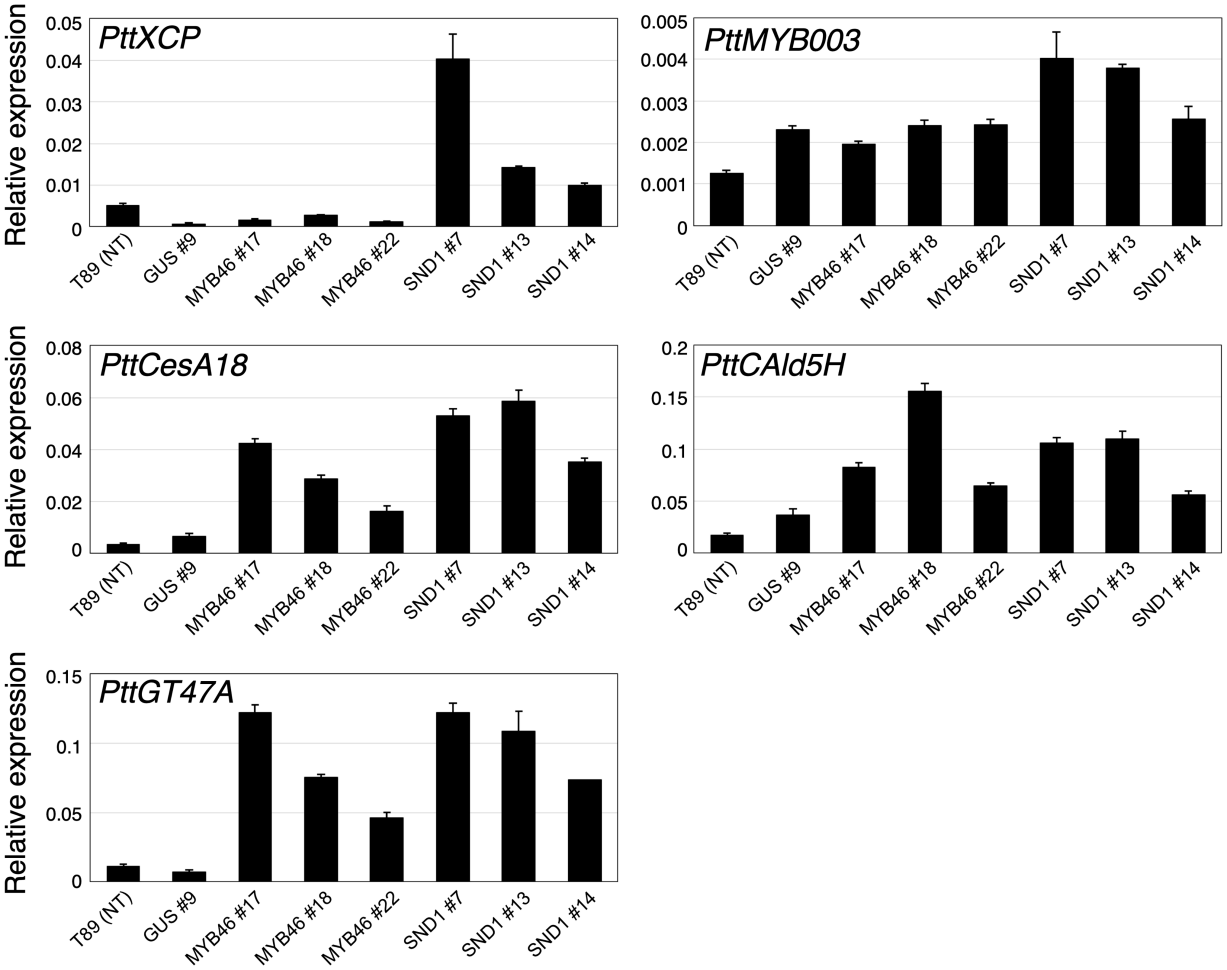
Expression levels of xylem-related genes in transgenic aspens. The *PttXCP1*, *PttMYB003*, *PttCesA18*, *PttCAld5H*, and *PttGT47A* mRNAs were quantified in non-transgenic (NT) aspen (T89), the *PtCesA18pro::GUS* (*GUS*) lines, the *PtCesA18pro::AtMYB46:VP16* (*MYB46*) lines, and the *PtCesA18pro::AtSND1:VP16* (*SND1*) lines using RT-qPCR. The expression levels of the analyzed genes were normalized against the expression level of *ELFA*. The data are displayed as means ± SD (*n* = 3).

Based on the expression levels of the transgenes (Figures 2E–G), we selected three lines of each transgenic plant for further detailed analysis. First, the expression levels of *Populus tremula × tremuloides XYLEM CYSTEINE PROTEASE 1* (*PttXCP1*), encoding a cysteine protease involved in PCD (Avci et al., 2008); *PttMYB003*, encoding a poplar *AtMYB46* homolog (Ohtani et al., 2011; Zhong et al., 2013); *PttCesA18*, encoding a xylem-specific cellulose synthase (Wu et al., 2000; Suzuki et al., 2006); *PttGT47A*, encoding an enzyme for hemicellulose biosynthesis; and *CONIFERALDEHYDE 5-HYDROXYLASE* (*PttCAld5H*), encoding an enzyme for monolignol biosynthesis (Hori et al., 2020) were examined using Reverse Transcription-Quantitative PCR (RT-qPCR) with the stem-derived total RNAs (Figure 3). *PttXCP1* and *PtMYB0003* were upregulated only in the *PtCesA18pro::AtSND1:VP16* plants (Figure 3), while the other SCW biosynthesis genes were upregulated in both the *PtCesA18pro::AtMYB46:VP16* and *PtCesA18pro::AtSND1:VP16* lines, relative to their expression in the non-transgenic plants and the vector control *PtCesA18pro::GUS* plants (Figure 3). This is consistent with the known transcriptional regulatory hierarchy, in which the VNS proteins, including AtSND1, can induce the expression of the secondary master switch *MYB* genes such as *AtMYB46* and *AtMYB83*, as well as the PCD-related and SCW-related genes, while the secondary master switch MYB proteins mainly upregulate SCW-related genes (Zhong et al., 2010a; Schuetz et al., 2013; Nakano et al., 2015; Ohtani and Demura, 2019; Kamon and Ohtani, 2021). We could not confirm the increased abundance of the proteins of AtVND7:VP16, AtSND1:VP16, nor AtMYB46:VP16 proteins, due to the absence of specific antibodies for these transcription factors. However, based on the increased expression of the downstream genes of AtSND1 and AtMYB46 in transgenic plants (Figure 3), we concluded that our system could successfully function as expected (Figure 1F).

### Secondary Xylem Phenotypes of *PtCesA18pro::AtMYB46:VP16* and *PtCesA18pro::AtSND1:VP16* Transgenic Plants

**Figure 4:**
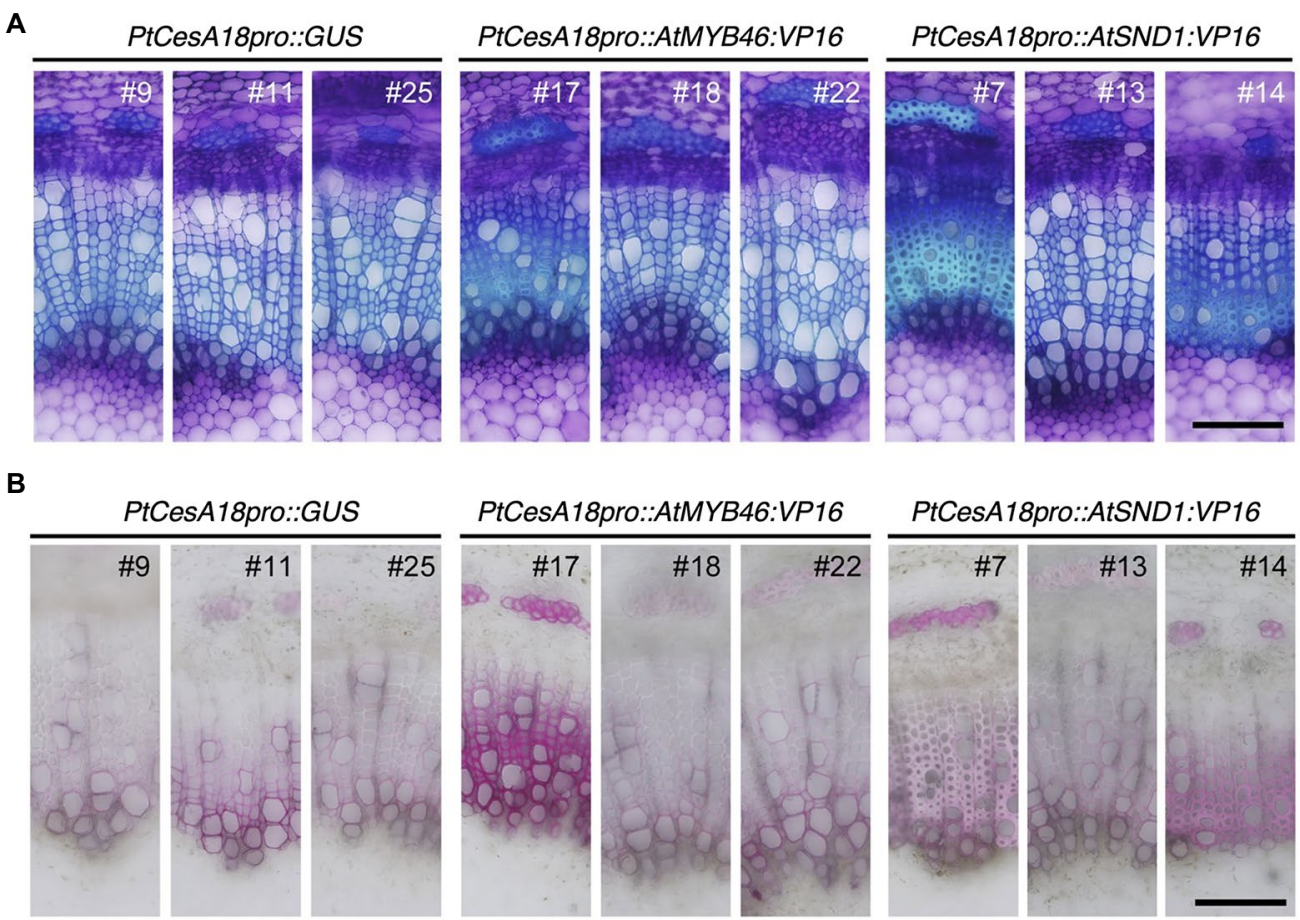
Histological observations of xylem tissues in transgenic aspens. **(A)** Stem sections of *PtCesA18pro::GUS*, *PtCesA18pro::AtMYB46:VP16*, and *PtCesA18pro::AtSND1:VP16* stained with toluidine blue. **(B)** Stem sections of *PtCesA18pro::GUS*, *PtCesA18pro::AtMYB46:VP16*, and *PtCesA18pro::AtSND1:VP16* stained with phloroglucinol-HCl for the detection of lignin. Bars = 100 μm.

Next, we examined the cell arrangement and SCW deposition in the secondary xylem of the *PtCesA18pro::AtMYB46:VP16* and *PtCesA18pro::AtSND1:VP16* transgenic plants (Figure 4). Toluidine blue staining showed that the size and cell arrangement of the xylem tissues of *PtCesA18pro::AtMYB46:VP16* lines 18 and 22 and *PtCesA18pro::AtSND1:VP16* line 13 were not significantly different from that of the control *PtCesA18pro::GUS* plants, whereas the width of the xylem was smaller in *PtCesA18pro::AtMYB46:VP16* line 17, which had a high *AtMYB46* expression level (Figure 2F), and in *PtCesA18pro::AtSND1:VP16* lines 7 and 14 (Figure 4A). In the xylems of *PtCesA18pro::AtMYB46:VP16* line 17 and *PtCesA18pro::AtSND1:VP16* lines 7 and 14, bright blue–stained cells were observed in the inner part of the secondary xylem and phloem fiber cells (Figure 4A). The bright blue signal by toluidine blue staining indicates lignin deposition (Pradhan Mitra and Loqué, 2014), thus we next used phloroglucinol, which stains lignin deposits red, to make clear lignin deposition additionally. The results showed strong red signals in the phloem fibers of *PtCesA18pro::AtMYB46:VP16* line 17 and *PtCesA18pro::AtSND1:VP16* lines 7 and 14 (Figure 4B). Lacking of bright blue toluidine blue signals and red phloroglucinol signals in *PtCesA18pro::AtSND1:VP16* line 13 indicated that no changes in lignification in the line 13. In addition, intense red signals and bright pink to white signals were observed in the xylem cells of *PtCesA18pro::AtMYB46:VP16* line 17 and *PtCesA18pro::AtSND1:VP16* lines 7 and 14, respectively, in comparison with the control plants (Figure 4B). The different coloration of phloroglucinol signals has been shown to reflect the different thickness of SCW and/or lignin S/G ratio (Pradhan Mitra and Loqué, 2014; Blaschek et al., 2020), and the bright pink to white phloroglucinol signals are observed when the SCW are thick or S-lignin rich SCWs are present (Blaschek et al., 2020). Thus, these observations suggested that SCW deposition, including lignification, was enhanced in these three lines, and that the acceleration of SCW deposition occurs simultaneously with a decrease in the size of the xylem regions.

**Figure 5:**
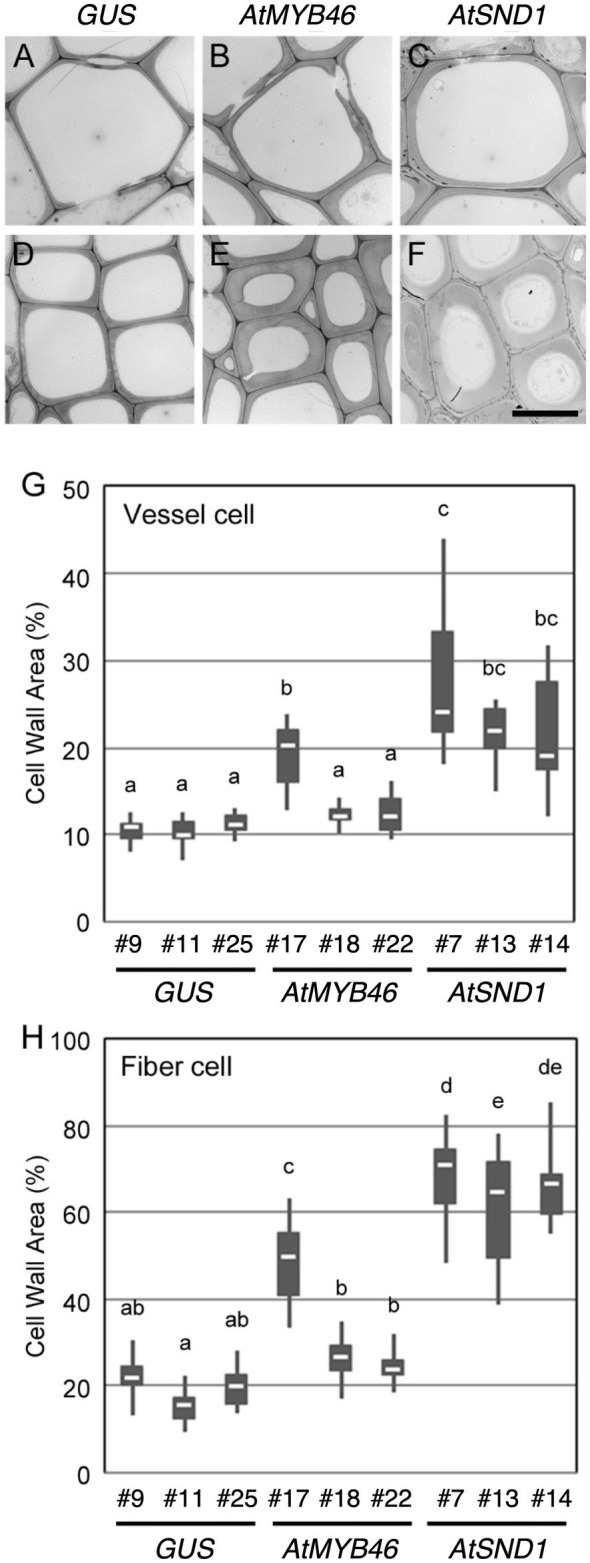
Cell wall thickness in the transgenic aspens. **(A–F)** Typical transmission electron microscope (TEM) images of xylem vessel cells **(A–C)** and xylem fibers cells **(D–F)** in the 10th elongating internodes of *PtCesA18pro::GUS* line 9 **(A,D)**, *PtCesA18pro::AtMYB46:VP16* line 17 **(B,E)**, and *PtCesA18pro::AtSND1:VP16* line 7 **(C,F)**. Bar = 10 μm. **(G,H)** Box plots of the cell wall area of the xylem vessel cells **(G)** and xylem fiber cells **(H)**. Cell wall area is shown as a ratio of the entire cell area. Different letters indicate statistically significant differences (Tukey–Kramer test: *p* < 0.05; *n* = 7–12 for xylem vessel cells, *n* = 14–29 for xylem fiber cells).

To examine the cell wall thickness of the secondary xylem cells, a TEM analysis was performed for both the xylem vessel cells (Figures 5A–C) and the xylem fiber cells (Figures 5D–F). In the *PtCesA18pro::AtMYB46:VP16* plants, the cell wall thickness of both the vessel and fiber cells was only statistically increased in line 7 compared with the controls (Figures 5G,H). By contrast, all three lines of *PtCesA18pro::AtSND1:VP16* showed a significant increase in the cell wall thickness of both the xylem vessel and fiber cells relative to the control plants (Figures 5G,H), despite the lack of clear changes of fiber cells in the line 13 by the histological observation (Figure 4). *PtCesA18pro::AtSND1:VP16* line 13 has a larger variation in cell wall thickness of fiber cells than the other two lines, line 7 and 14 (Figure 5H), thus the effects of cell wall thickness could be unstable in the line 13. Notably, the effect of the high transgenic *AtSND1* expression on cell wall thickness was greater than that of *AtMYB46* transgenic expression (Figures 5G,H), and in the case of the *PtCesA18pro::AtMYB46:VP16* plants, the increase in cell wall thickness was detected only in line 17, which had the highest *AtMYB46* expression level (Figure 2F). Such differences in overexpression effects might reflect the difference of molecular function between AtSND1 and AtMYB46; AtSND1 activates the entire molecular program of fiber cell differentiation, including not only SCW biosynthesis enzymes but also the other regulatory factors, such as secretion machinery (Mitsuda et al., 2005, 2007; Zhong et al., 2006, 2010b; Ohashi-Ito et al., 2010), while the regulatory targets of AtMYB46 are mainly SCW-related enzymatic genes (Zhong et al., 2007; Ko et al., 2009; McCarthy et al., 2009).

### Wood Cell Wall Properties in *PtCesA18pro::AtMYB46:VP16* Transgenic Plants

**Figure 6:**
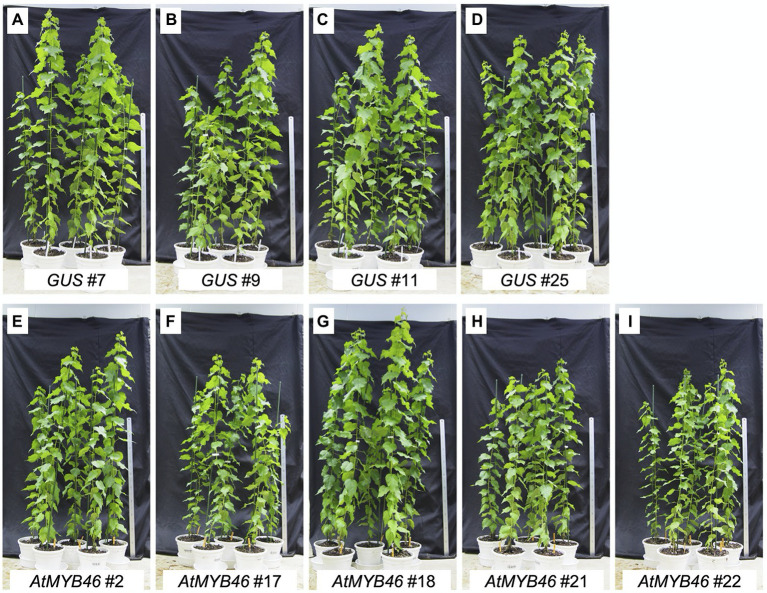
Growth and morphology of the *PtCesA18pro::AtMYB46:VP16* plants grown in pots of soil in the greenhouse. Phenotypes of the transgenic plants *PtCesA18pro::GUS* (vector control; **A–D**) and *PtCesA18pro::AtMYB46:VP16* **(E–I)** 3 months after being transferred from the sterile half-strength Murashige and Skoog growth medium to pots of soil. Scale = 1 m.

The transgenic aspens grown in the sterile MS growth medium showed enhanced SCW deposition in their secondary xylem (Figures 4, 5). To further assess the wood structure and wood cell wall properties, we tried to grow the transgenic plants in pots of soil in the greenhouse (Figure 6). Five plants of each transgenic line (*PtCesA18pro::AtMYB46:VP16* and *PtCesA18pro::AtSND1:VP16*) were prepared and transferred into the soil. Unfortunately, no *PtCesA18pro::AtSND1:VP16* plants were able to survive being transplanted into the pots of soil. It was previously reported that the transgenic aspen expressing *OsSWN1:VP16*, a chimeric gene comprising a rice homolog of *AtSND1* and *VP16*, under the control of the *AtSND1* promoter showed no growth defects in the pots of soil (Sakamoto et al., 2016); thus, the lack of viability of our self-reinforced system with *PtCesA18pro::AtSND1:VP16* plants expressing *AtSND1* is likely caused by some factors that would be affected only in our self-reinforced system, but not in the traditional overexpression. Previously, we observed that cell wall modification actively occurred during the stress response in poplar (Chen and Polle, 2010; Janz et al., 2012; Polle et al., 2019; Hori et al., 2020), and the expression levels of the *VNS* genes, especially the poplar *SND1* homologs, were decreased in response to stresses in poplar (Hori et al., 2020), suggesting that the regulation of VNS activity could be crucial for the plant stress response. Based on this idea, we hypothesize that the *PtCesA18pro::AtSND1:VP16* plants cannot perform the proper acclimatization processes required for the transfer from humid aseptic growth medium containers to relatively dry soils in the greenhouse.

In contrast, the *PtCesA18pro::AtMYB46:VP16* plants and the vector control plants continuously grew in the greenhouse pots of soil, reaching ~1.5 m in height during the 3-month culture (Figure 6). The height and morphology of the *PtCesA18pro::AtMYB46:VP16* plants were comparable to the vector control plants, suggesting no significant growth effect of the high transgenic *AtMYB46* expression in our self-reinforced system. These results indicated that the current version of the self-reinforced system with the *VNS* genes is not suitable for directly obtaining plant biomass for practical use, but that the self-reinforced system using the *AtMYB46* gene has potential for further practical use.

**Figure 7:**
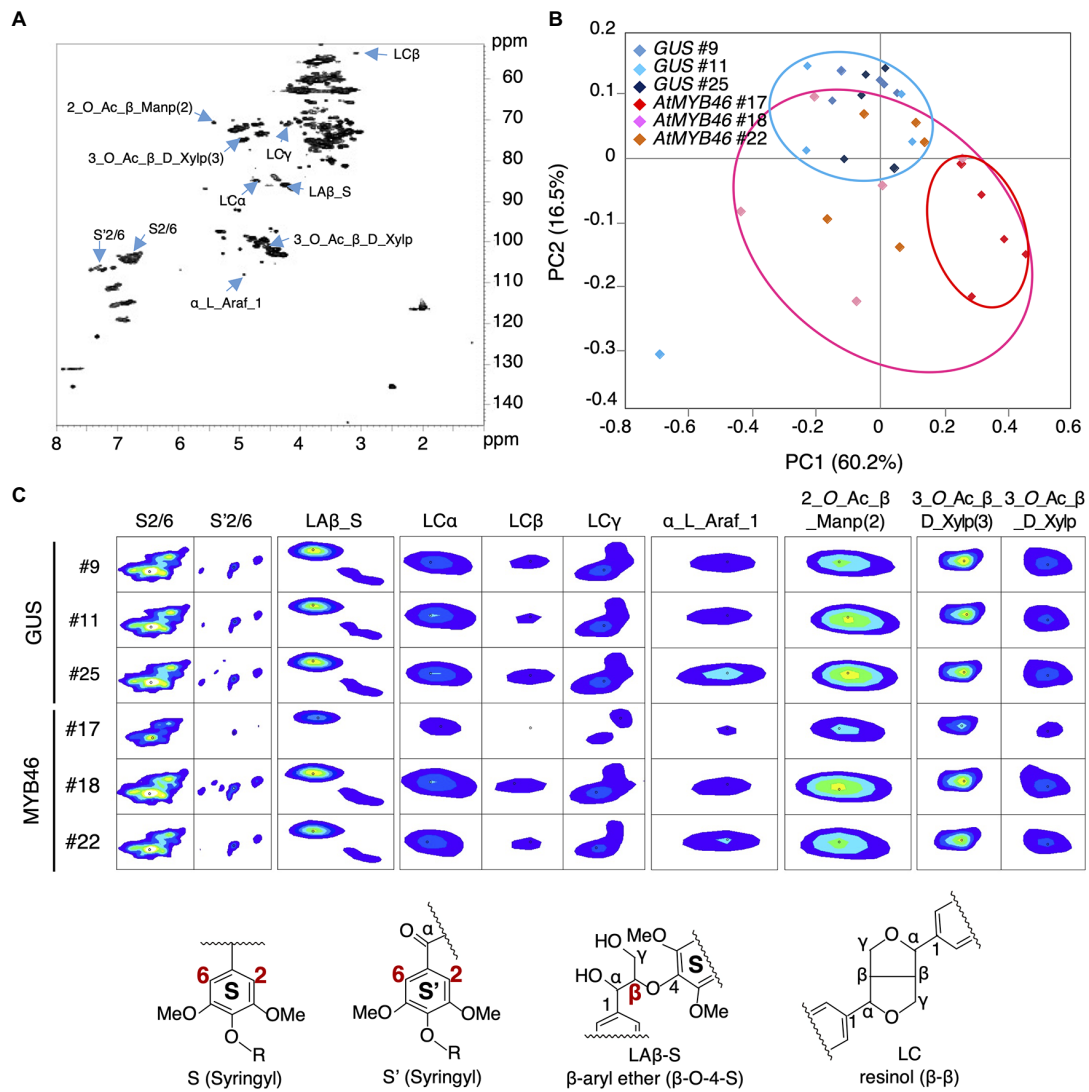
Nuclear magnetic resonance (NMR) footprint analysis of stem samples of the transgenic aspens. **(A)** Typical hetero-nuclear single quantum coherence (HSQC) image of the stem samples. The peaks whose signals were decreased in *PtCesA18pro::AtMYB46:VP16* line 17 are indicated with right blue arrows. **(B)** Principal component analysis (PCA) plots of the *PtCesA18pro::GUS* (vector control) and *PtCesA18pro::AtMYB46:VP16* stem samples. Blue, pink, and red ovals indicate the distribution ranges of the *PtCesA18pro::GUS* samples, all *PtCesA18pro::AtMYB46:VP16* samples, and the samples specifically from *PtCesA18pro::AtMYB46:VP16* line 17, respectively. **(C)** NMR signal peaks that were significantly decreased in *PtCesA18pro::AtMYB46:VP16* line 17. Representative structures for lignin-related NMR peaks are shown at the bottom. For each line, five individuals were examined as biological replicates.

Next, the cell walls of the *PtCesA18pro::AtMYB46:VP16* stems (line 17, 18, and 22) were assessed by NMR footprinting analysis (Kim and Ralph, 2010; Mansfield et al., 2012; Hori et al., 2020; Akiyoshi et al., 2021). The cell wall fractions were extracted from the stem samples of transgenic plants using a DMSO-*d*_6_/pyridine-*d*_5_ solution, then subjected to the HSQC NMR analysis, which has proven to be an effective method to examine the polysaccharides and lignin components in unfractionated plant cell wall materials (Kim and Ralph, 2010; Mansfield et al., 2012; Tobimatsu et al., 2013; Kim et al., 2017; Zhang et al., 2019; Figure 7A). We performed the principal component analysis (PCA) of the signal intensities of the NMR peaks. The PCA plot demonstrated that the cell wall properties of *PtCesA18pro::AtMYB46:VP16* woods could be differentiated from that of the vector control plants (Figure 7B); the *PtCesA18pro::AtMYB46:VP16* samples were generally located in the regions where PC2 takes a negative value (see pink circle in Figure 7B). The samples of *PtCesA18pro::AtMYB46:VP16* line 17 in particular were clustered in the region with positive PC1 values and negative PC2 values (see red circle in Figure 7B), suggesting relatively large changes in cell wall composition in this line.

**Figure 8:**
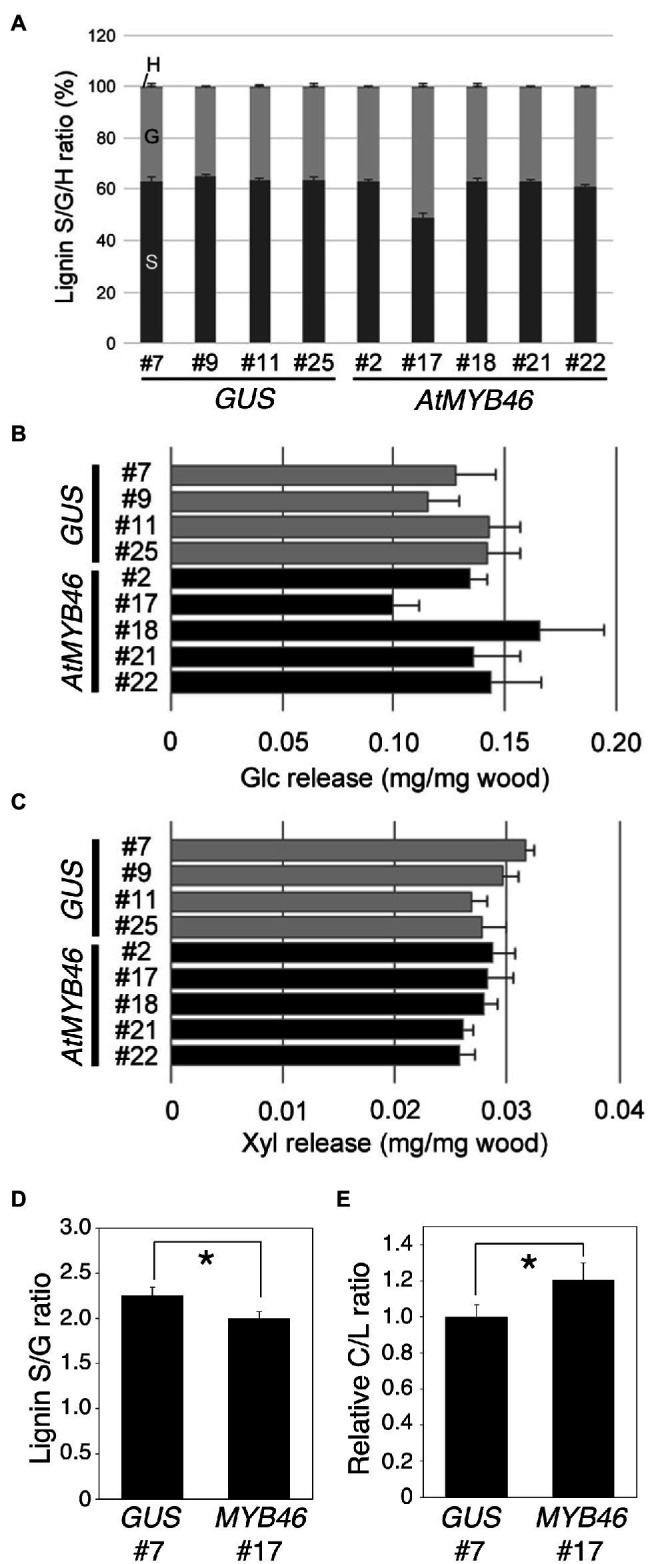
Lignin S/G ratio and enzymatic saccharification efficiency of transgenic aspens. **(A)** Lignin S/G ratio of the *PtCesA18pro::GUS* (vector control) and *PtCesA18pro::AtMYB46:VP16* stem samples. The data are shown as means ± SD (*n* = 5). **(B,C)** Sugar release from the *PtCesA18pro::GUS* (vector control) and *PtCesA18pro::AtMYB46:VP16* stem samples after enzymatic treatment. The amounts of glucose (Glc; **B**) and xylose (Xyl; **C**) obtained from the treated wood samples are shown (means ± SD; *n* = 5). **(D,E)** Pyrolysis-GC/MS analysis of *PtCesA18pro::GUS* line 7 and *PtCesA18pro::AtMYB46:VP16* line 17. The lignin S/G ratio **(D)** and carbohydrate:lignin (C/L) ratio (E) were shown (*n* = 3). For the C/L ratio, the average value of *PtCesA18pro::GUS* line 7 data was set as 1.0, and all results were shown as relative values. Asterisk indicates statistically significant difference (Student’s *t* test, *p* < 0.05).

We further examined the peaks that contributed to the PC1 score in Figure 7B, and found that such peaks contained many lignin-related NMR peaks (Figures 7A,C). In *PtCesA18pro::AtMYB46:VP16* line 17, the signal intensities of the NMR peaks corresponding to syringyl (S) lignin (S2/6 and S’2/6), β-aryl ether (Laβ_S), and resinol (LCα, LCβ, and LCγ) were significantly reduced (Figure 7C), suggesting that the S-lignin monomer and related structures would be decreased in line 17. Indeed, the S/G ratio of lignin was about 1.0 in this line (Figure 8A), which is lower than the typical S/G ratio of poplar wood (1.8–2.3; Davison et al., 2006). In Arabidopsis mature stems, the S/G ratio is around 0.4 (Sibout et al., 2005); thus, the high transgenic expression of Arabidopsis *MYB46* might influence the lignin biosynthesis process in the transgenic hybrid aspen, enabling the biosynthesis of lignin with intermediate characters between poplar and Arabidopsis. In addition to lignin-related NMR peaks, sugar-related peaks also showed decreased signal intensities in the *PtCesA18pro::AtMYB46:VP16* line 17 (Figure 7C). The intensity of the signal related to arabinose (α_L_Ara*f*_1), which is largely contained in the primary cell wall, was decreased, possibly due to the enhancement of SCW formation in line 17 (Figure 5). Interestingly, the signals related to acetylated hemicellulosic components, such as acetylated mannan [2-*O*-Ac-β-D-Man*p* (2)] and acetylated xylan [3-*O*-Ac-β-D-Xyl*p* and 3-*O*-Ac-β-D-Xyl*p* (3)], were also reduced in line 17 (Figure 7C); therefore, in line 17, the inhibition of hemicellulose acetylation and/or the deacetylation of hemicellulose is enhanced.

The lignin S/G ratio and degree of hemicellulose acetylation are critical factors for the regulation of SCW properties and the enzymatic saccharification of wood biomass (Chen and Dixon, 2007; Studer et al., 2011; Pawar et al., 2013, 2017), and the NMR data showed a decreased S/G ratio in *PtCesA18pro::AtMYB46:VP16* line 17 (Figure 8A). Thus, the enzymatic saccharification efficiency of *PtCesA18pro::AtMYB46:VP16* was examined (Figures 8B,C). No statistically significant differences in the release of glucose or xylose were detected between *PtCesA18pro::AtMYB46:VP16* and the vector control *PtCesA18pro::GUS* plants (Figures 8B,C). Since the SCW accumulation was enhanced in *PtCesA18pro::AtMYB46:VP16* line 17 (Figure 5), this finding of saccharification efficiency seemed to be a discrepancy; however, in the *PtCesA18pro::AtMYB46:VP16* line 17, lignin deposition may have been increased (Figure 4B) and the S/G ratio was decreased (Figure 8A). High amounts of lignin with a low S/G ratio can decrease the saccharification efficiency (Studer et al., 2011); therefore, it is possible that the accumulation of SCW material in *PtCesA18pro::AtMYB46:VP16* line 17 increased its recalcitrance for sugar release, offsetting the effects of enhanced SCW accumulation to result in no difference in enzymatic saccharification efficiency (Figure 8C). To make clear this point, we performed the pyrolysis-GC/MS analysis of cell wall extracts (Figures 8D,E). The pyrolysis-GC/MS analysis confirmed the lower S/G ratio in the *PtCesA18pro::AtMYB46:VP16* line 17 (Figure 8D). It was also indicated that the carbohydrate portion to lignin was increased in the *PtCesA18pro::AtMYB46:VP16* line 17 (Figure 8D), suggesting that the *MYB46* overexpression would not increase the lignin contents, but possibly change the balancing between carbohydrates and lignin in the enhanced SCW. Taken together, our data demonstrated the basic effectiveness of our SCW-related transcription factor–based self-reinforced system for enhanced SCW accumulation (Figures 4, 5) and the modification of SCW properties (Figures 7, 8). However, the current system could not increase sugar yields through enzymatic saccharification (Figures 8B,C), suggesting that we must further improve the design of this self-reinforced system.

## Conclusion and Perspectives

In this work, we tested our idea of a SCW-related transcription factor–based self-reinforced system in the useful woody plant *Populus*. The results indicated that this system can work to enhance SCW accumulation in hybrid aspen without visible difference of plant growth, clearly indicating the effectiveness of this system for increasing biomass production per space; however, we also recognized that the selection of transcription factors is critical for this system. The strong boosting of VNS protein activity is not suitable for the self-reinforced system, since the expression of *VNS* genes would be tightly regulated to generate proper xylem cells in response to environmental conditions. Indeed, our results demonstrated that the transgenic aspens carrying *PtCesA18pro::AtVND7:VP16* or *PtCesA18pro::AtSND1:VP16* cannot survive well. By contrast, *PtCesA18pro::AtMYB46:VP16* successfully functioned to enhance SCW accumulation in the xylem cells of the transgenic aspens, as expected (Figures 4, 5). Enhanced SCW accumulation was observed only in *PtCesA18pro::AtMYB46:VP16* line 17, which showed the highest expression of the transgene *AtMYB46* (Figure 2F), and even in this line, the yields of glucose and xylose following enzymatic saccharification were no different to the control lines (Figures 8B,C). These results suggest that there is still much room for improvement in the selection of transcription factors for this self-reinforced system.

Interestingly, the RT-qPCR results of the *PtCesA18pro::AtSND1:VP16* and *PtCesA18pro::AtMYB46:VP16* expression levels showed that the degree of enhancement of SCW deposition did not correlate well with the expression levels of the tested SCW-related genes, which are all well-known SCW-related factors working downstream of the VNS module (Figures 3–5). Considering that the heterologous *VNS* genes induce the accumulation and/or modification of the SCW more effectively than endogenous *VNS* genes (Ohtani et al., 2011; Sakamoto et al., 2016; Akiyoshi et al., 2021), genes unexpectedly related to SCW accumulation could be affected in *PtCesA18pro::AtMYB46:VP16* line 17. These differences in target genes may be related to the differences in the cis-sequence binding properties of the VNS proteins from each plant species, as well as the differences in their interactivity with other proteins. Thus, we have to consider the endogenous molecular mechanisms of SCW formation in hybrid aspen more carefully, and identify the critical points for further engineering of woody biomass. To improve the design of the SCW-related transcription factor–based self-reinforced system for the further enhancement of woody biomass utilization, we should further analyze the characteristics of the VNS and MYB proteins as transcription factors, especially focusing on their functionality in target plant species for the engineering.

## Data Availability Statement

The original contributions presented in the study are included in the article/**Supplementary Material**; further inquiries can be directed to the corresponding authors.

## Author Contributions

YN, MO, and TD designed the experiments. YN, AI, TM, and MO grew, collected, and sampled the material. YN, HE, CH, and MO performed the microscope and TEM observations, gene expression analysis, and saccharification test. LG and MS performed pyrolysis-GC-MS analysis. TM and JK designed and performed the NMR analyses. YN and MO wrote the manuscript. All authors contributed to the article and approved the submitted version.

## Funding

This work was in part supported by the RIKEN Center for Sustainable Sciences, MEXT KAKENHI (JP18H05484 and JP18H05489 to MO and TD, and JP20H05405 and JP21H05652 to MO, Grants-in-Aid from the NC-CARP project to YN, HE, and TD), JSPS KAKENHI (JP20H03271 to MO and JP18H02466 to TD), and ERATO JST (JPMJER1602 to MO).

## Conflict of Interest

The authors declare that the research was conducted in the absence of any commercial or financial relationships that could be construed as a potential conflict of interest.

## Publisher’s Note

All claims expressed in this article are solely those of the authors and do not necessarily represent those of their affiliated organizations, or those of the publisher, the editors and the reviewers. Any product that may be evaluated in this article, or claim that may be made by its manufacturer, is not guaranteed or endorsed by the publisher.

## Footnotes

^1^ https://imagej.nih.gov/ij/

^2^ https://www.R-project.org
