# Supplementary material for: Enhancement of Secondary Cell Wall Formation in Poplar Xylem Using a Self-Reinforced System of Secondary Cell Wall-Related Transcription Factors
Source: Front Plant Sci. 2022 Mar 14;13:819360. doi: 10.3389/fpls.2022.819360 (PMC8967175; doi:10.3389/fpls.2022.819360)
Supplement: Supplementary file 1 [file Data_Sheet_1.PDF]

Supplementary Material

1 Supplementary Figure and Table

1.1 Supplementary Figure

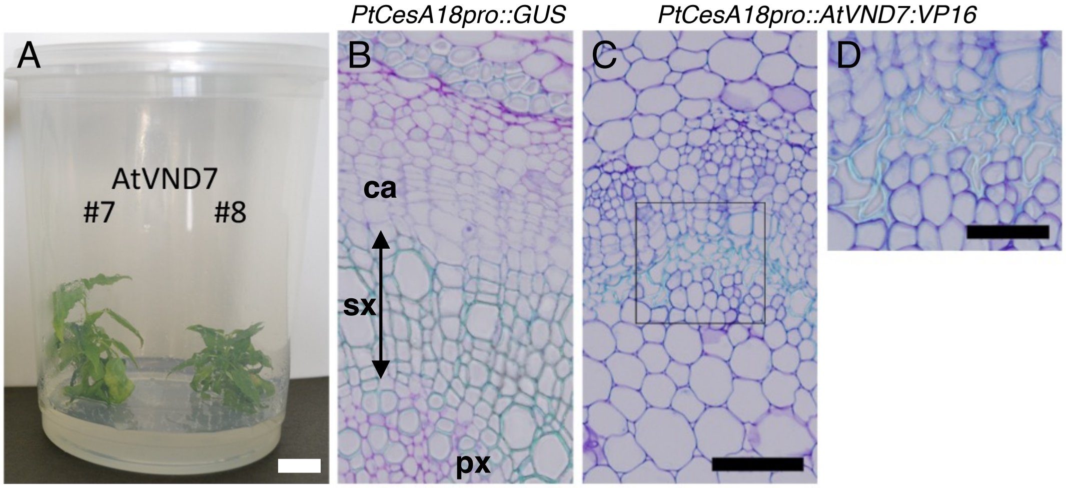

Supplementary Figure 1. Histological observations of xylem tissues in *PtCesA18pro::AtVND7:VP16* aspens. (A) Transgenic aspens grown in a sterile half-strength Murashige and Skoog growth medium. Typical view of plants carrying the *PtCesA18pro::VND7:VP16* line 7 and 8. **(B,C)** Stem sections of *PtCesA18pro::GUS* **(B)** and *PtCesA18pro::AtVND7:VP16* **(C)** stained with toluidine blue. **(D)** Enlargement of the area indicated by the black square is shown in **(C)**. px; primary xylem, sx; secondary xylem, ca; cambium. Bars = 2 cm (A), 50 µm (B,C), 25 µm (D). In *PtCesA18pro::AtVND7:VP16* xylem, no clear secondary xylem was not observed. Instead, the collapsed xylem vessel-like cells could be recognized at the position where secondary xylem should be found.

1.2 Supplementary Table

Supplementary Table 2. Primers used in this study.

**For cloning**		
PtCesA18p-Hind_F	aagcttaactagttcagtttttacaccgttttgg	PtCesA18pro for GUS
PtCesA18p-Sma_R2	cccgggCAGATTCCATCATcttgcttattaccactt	PtCesA18pro for GUS
PtCesA18p-EV_F	gatatcaaggcttaactagttcagtttttacaccg	PtCesA18pro for SND1, VND7, MYB46
PtCesA18p-SND1_R	CTTATTATCAGCCATcttgcttattaccac	PtCesA18pro for SND1
PtCesA18p-SND1_F	gtggtaataagcaagATGGCTGATAATAAG	SND1
SND1(-stop)_Avr_R	cctaggTACAGATAAATGAAGAAGTGGGTC	SND1
PtCesA18p-VND7_R	CATTATATTATCCATcttgcttattaccac	PtCesA18pro for VND7
PtCesA18p-VND7_F	gtggtaataagcaagATGGATAATATAATG	VND7
VND7(-stop)_Avr_R	cctaggCGAGTCAGGGAAGCATCCAAGAGA	VND7
CesA18p-MYB46_R	CTCTGGCTTCCTCATcttgcttattaccac	PtCesA18pro for MYB46
CesA18p-MYB46_F	gtggtaataagcaagATGAGGAAGCCAGAG	MYB46
MYB46(-stop)Avr_R	cctaggTATGCTTTGTTTGAAGTTGAAGTAAA	MYB46
**For real-time PCR**		
PtELF4A uni	ACACAGTCTCAGCTACTCATGGTGA	
PtELF4A rev	ATTTATGACAAGGGACACTTGCTG	
VP16 R1	ACTCGAAGTCGGCCATATCCAGAG	
MYB46 RT F	CCTTGACCCACATACAAACCAACAA	
SND1 RT F	AGTTTCCTGACTCCAAGCAAACTCG	
PttXCP RT-F	CGAAGACTTCAGTTACAGAGATGTGG	
PttXCP RT-R	CACCGTTGTTGACTATGAACTCG	
PttMYB003 RT-F	CCACATCCTCACCAAACGATAGTAC	
PttMYB003 RT-R	CGCCACTCACATCATATCGGTTA	
PttGT47A RT-F	CACTTGTGATCTCACACCCATG	
PttGT47A RT-R	CCAAAGTAGAACGCTGGAGTAGC	
PttCAld5H RT-F	CCAATATAGGCAAGCCTGTGAATC	
PttCAld5H RT-R	GATAGAGTCGATGAATCTATCAAGAGCC	
PttCesA18 RT-F	GGAATCCTGTCAATAGAGAAACGTA	
PttCesA18 RT-R	CAGAGATTCAAATGAAAGCATAGCT	
GUS-for	TTGATGTGCTGTGCCTGAACC	
GUS-rev	CCCTTTCTTGTTACCGCCAAC	
**For genome check**		
PtCesA18p-VND7_F	gtggtaataagcaagATGGATAATATAATG	
PtCesA18p-SND1_F	gtggtaataagcaagATGGCTGATAATAAG	
CesA18p-MYB46_F	gtggtaataagcaagATGAGGAAGCCAGAG	
VP16+stop_R	TCAgCTAcTTACCCACCGTACTCGTCAATTCC	
Bgl-NPT_F	agatctatgattgaacaagatggattg	
NPT-Bgl_R	agatctcagaagaactcgtcaagaag	
